# The Medical Profession and the War Office

**Published:** 1917-05-05

**Authors:** 


### The Hospital

The Workers' Newspaper of Administrative Medicine and Institutional Life, Administration, National Insurance and Health.

No. 1613, Vol. LXII. SATURDAY, MAY 5, 1917. PRICE ONE PENNY

### THE MEDICAL PROFESSION AND THE WAR OFFICE.

It has been our continuous endeavour since the outbreak of war to support in these columns the actions of the authorities charged with the heavy responsibility of the nation's interests. We have not always approved these actions, and we have sometimes felt it our duty to say so. But we have always recognised the difficulties of the situation and the patriotic motives which have prompted decisions; and when policy has once been authoritatively defined we have urged its acceptance. Regarding the special position of the medical profession in relation to the national cause we have even incurred the censure of some of our *confrères* by approving of mobilisation of the profession apart from the application of compulsion to other sections of the community. To this last-mentioned proposal, however, we attached certain conditions, and one of these was that the direction and organisation of such an enterprise should be left in the hands of a professional committee. Hence we make no new departure and develop no new critical attitude when we express, as we now do, a severely adverse judgment on the recent action of the War Office in calling to the Colours all medical men of military age without prior consultation with the Central Medical War Committee and the Committee of Reference of the two Royal Colleges. This summons, if put into effective operation, would have left certain towns and districts of the country practically denuded of medical practitioners, with results which it is unnecessary to particularise. That the authorities acting under the Military Service Acts had the legal right to take this step we do not question. At least they had the right to compel the practitioners in question to join the Army, but whether they had the right to claim professional service is another question. Anyhow there was the plain announcement that all doctors whose age brought them under the Military Service Acts were to hold themselves at the disposition of the authorities, and to this as a legal position we make no challenge. Upon the policy and advisability and justice of the step there is, however, something to be said.

The actual position prior to the new departure of a week or so ago was that for nearly two years doctors in thousands have been supplied to the Army through the activities of the professional committees mentioned above. These committees as the result of much labour and expense have surveyed the whole field of medical service with a view to meet so far as possible the needs both of the Army and the civilian population, and it is largely due to their efforts that many practitioners above the military age have accepted commissions in the R.A.M.C. There is no other organisation in existence which knows what more is possible in respect to the distribution of medical service to the Army on the one hand, and to the general public on the other. Yet, exercising its technical rights, the War Office, which has been helped again and again by the professional committees, ignores them at a critical moment and takes a decision which, if adopted in practice, would have had most harmful effects, seeing that it would have deprived various areas of the country of anything like an adequate supply of practitioners. It is little wonder that in these circumstances the medical committees felt themselves compelled publicly to dissociate themselves from all further responsibility in the matter. They have laboured without reward and with much individual sacrifice to meet a difficult situation, and their work has been of high value to the nation. As a result of this work there has followed the conviction that in their hands lay the arrangement of the medical service available both for the Army and for the general community, and it was impossible for them to remain silent when their authority was abruptly pushed to one side. The course they took was both dignified and necessary, and it is to their protest that we owe the re-establishment of a relatively sound position. The authorities have so far yielded that the committees feel justified in continuing their work, but the experience is a significant one and cannot be ignored. It must be recorded as an event which shows plainly the need for the existence of an organised force to protect the medical necessities of the home population against unfair and unreasonable demands. The medical profession has no need to protest its patriotism, but its willing service must be contributed according to knowledge and not according to the dictates of unenlightened and arbitrary authority. Where the exact responsibility for the recent disregard of the professional committees lies we will not trouble to inquire. But we sincerely trust that it does not lie with the heads of the Army Medical Service, for these gentlemen have full knowledge of the contribution which has been offered by their civilian colleagues and of the loyal and sustained efforts which have been made to secure an efficient service for our fighting men. These efforts will be continued, but it must be plainly recognised that responsibility cannot be accepted if authority at some selected moment is to be denied. There is perhaps a general lesson in the story. Among us are some who cultivate an admiration for the proposal to organise the activities and destinies of the profession under the guidance and control of a State Department. What this may involve has a graphic display in the experience which we here recite. The moral is so clear that it can hardly be missed, and we shall keep it for quotation as occasion arises.

